# Supplementary material for: Mathematically modeling spillovers of an emerging infectious zoonosis with an intermediate host
Source: PLoS One. 2020 Aug 26;15(8):e0237780. doi: 10.1371/journal.pone.0237780 (PMC7449412; doi:10.1371/journal.pone.0237780)
Supplement: S1 Appendix — (PDF) [file pone.0237780.s001.pdf]

Theorem 1. *There is only one admissible endemic equilibrium E_e^d in the domestic compartment. At this equilibrium, we have $S_d^* < \min\{b_d/(m_d + p_d I_w^*), (\gamma_d + m_d)/\beta_d\}$.*

Proof. Adding the equations for $\frac{dS_d}{dt}$, $\frac{dI_d}{dt}$, and $\frac{dT_d}{dt}$, we obtain

$$b_d - m_d S_d^* - (\gamma_d + m_d) I_d^* - (\gamma_d + m_d) T_d^* = 0. \qquad (\star_1)$$

Further, from the equation for $\frac{dT_d}{dt}$, we isolate

$$I_d^* = \frac{1}{\mu}(\gamma_d + m_d - \beta_d S_d^*) T_d^*. \qquad (\star_2)$$

Substituting $\star_2$ into $\star_1$, we get

$$T_d^* = \frac{b_d - m_d S_d^*}{(\gamma_d + m_d) + \frac{1}{\mu}(\gamma_d + m_d)(\gamma_d + m_d - \beta_d S_d^*)}. \qquad (\star_3)$$

Since the quantities I_d^* and T_d^* are both nonnegative, it follows immediately that the equilibrium value S_d^* must have a natural upper bound:

$$S_d^* \le \frac{\gamma_d + m_d}{\beta_d} \le \frac{b_d}{m_d}. \qquad (1)$$

Hence we can confirm the denominator in ($\star_3$) is strictly positive.

We also note that from the equation for $\frac{dR_d}{dt}$, we have $R_d^* = \frac{\gamma_d(I_d^* + T_d^*)}{m_d}$. We thus calculate the equilibrium values S_d^* by substituting $\star_2, \star_3$ into the equation for $\frac{dS_d}{dt}$ and obtain, after rearranging:

$$\begin{aligned} b_d - p_d S_d^* I_w^* - m_d S_d^* = \beta_d S_d^*(I_d^* + T_d^*) &= \beta_d S_d^*(1 + \frac{1}{\mu}(\gamma_d + m_d - \beta_d S_d^*)) T_d^* \\ &= \frac{\beta_d S_d^*(1 + \frac{1}{\mu}(\gamma_d + m_d - \beta_d S_d^*))(b_d - m_d S_d^*)}{(\gamma_d + m_d) + \frac{1}{\mu}(\gamma_d + m_d)(\gamma_d + m_d - \beta_d S_d^*)} \\ &= \frac{\beta_d S_d^*(b_d - m_d S_d^*)}{(\gamma_d + m_d)}. \end{aligned} \qquad (2)$$

We note that in the last step of the derivations above we cancel out the common factor $1 + \frac{1}{\mu}(\gamma_d + m_d - \beta_d S_d^*) > 0$, which is guaranteed by the inequality (1). It is easy to observe that there exist at most two possible equilibrium values of S_d^* as the roots of the quadratic equation (2), denoted by $S_{d_(1)}^* < S_{d_(2)}^*$, a consequence of our assumption that the transmission and recovery rates of both strains in domestic animals are equal.

We now proceed to prove only the smaller root $S_{d_(1)}^*$ is admissible for the long-term disease dynamics should there be nonzero disease burden (i.e., $I_d^* > 0$ and $T_d^* > 0$) in the domestic compartment. In fact, we can view S_d^* as the fixed point(s) satisfying

$$f(x) = g(x),$$

where $f(x)$ is a simple linear function, given by

$$f(x) = b_d - (p_d I_w^* + m_d) x,$$

and $g(x)$ is a quadratic function, given by

$$g(x) = \frac{\beta_d x (b_d - m_d x)}{(\gamma_d + m_d)}.$$

We can show that $f(0) = b_d > 0 = g(0)$, $f(b_d/(m_d + p_d I_w^*)) = 0 < g(b_d/(m_d + p_d I_w^*))$, and $0 > f(x) > g(x)$ for sufficiently large x. Furthermore, as both f and g are smooth continuous functions, according to the intermediate value theorem, there must exist two fixed points satisfying $f(x) = g(x)$, $S^*_{d_{(1)}} \in (0, b_d/(m_d + p_d I_w^*))$ and $S^*_{d_{(2)}} \in (b_d/(m_d + p_d I_w^*), \infty)$.

We have $b_d - p_d S_d^* I_w^* - m_d S_d^* = \beta_d S_d^*(I_d^* + T_d^*) > 0$, for nonzero disease burden $I_d^* > 0, T_d^* > 0$. Hence we must have $S_d^* < b_d/(m_d + p_d I_w^*)$. So we complete our proof that only the smaller root $S^*_{d_{(1)}}$ is admissible as the unique endemic equilibrium in the domestic compartment.

□

In line with our proof above, it is easy to directly check the discriminant of the quadratic equation in (2) is positive:

$$\Delta = [\beta_d b_d + (\gamma_d + m_d)(p_d I_w^* + m_d)]^2 - 4\beta_d m_d b_d(\gamma_d + m_d) > 0.$$

Then we obtain the endemic equilibrium for $S_d^* = S^*_{d_{(1)}}$:

$$S^*_{d_{(1)}} = \frac{\beta_d m_d + (\gamma_d + m_d)(p_d I_w^* + m_d) - \sqrt{\Delta}}{2\beta_d m_d}.$$

We have thus shown that, in the domestic compartment, there is one unique endemic equilibrium $E_e^d = (S_d^*, I_d^*, T_d^*, R_d^*)$, where

$$\begin{aligned} S_d^* &= S^*_{d_{(1)}}, \\ T_d^* &= \frac{b_d - m_d S_d^*}{(\gamma_d + m_d) + \frac{1}{\mu}(\gamma_d + m_d)(\gamma_d + m_d - \beta_d S_d^*)}, \\ I_d^* &= \frac{1}{\mu}(\gamma_d + m_d - \beta_d S_d^*)T_d^*, \\ R_d^* &= \frac{\gamma_d(I_d^* + T_d^*)}{m_d}. \end{aligned}$$
