# Supplementary material for: Mathematically modeling spillovers of an emerging infectious zoonosis with an intermediate host
Source: PLoS One. 2020 Aug 26;15(8):e0237780. doi: 10.1371/journal.pone.0237780 (PMC7449412; doi:10.1371/journal.pone.0237780)
Supplement: Attachment — Submitted filename: Review_PLOS_ONE_2020.pdf [file pone.0237780.s003.pdf]

Review: Mathematically modeling spillovers of an emerging infectious zoonosis with an intermediate host
Authors: Katherine Royce and Feng Fu
Journal: PLOS ONE

A mathematical model for a zoonosis is studied and applied to avian influenza. The model consists of three different populations, wild reservoir, intermediate domestic animals, and humans. The infection is spread from the wild to intermediate to humans. Spread to humans only occurs after a mutation of the virus in the intermediate host. The introduction is well written and the concept is interesting.

Unfortunately, the analysis is simplistic and incorrect. As written it is difficult to follow what is shown in the large number of Figures 4-13 in the simulations. The reproductive numbers for each population are not provided. The model is closely related to a multigroup model with two types of infection in the intermediate group.

The value of R_0 can be calculated at the disease-free equilibrium (dfe) by applying Theorem 2 in [37] as in lines 390-395. In addition, if the conditions (A1) and (A5) hold in [37], then Theorem 2 can be applied to show that the dfe is locally asymptotically stable. Theorems 1-5 and the lemmas are not required. The analyses for each population, Wild, Domestic, and Human, are trivial and are not needed when considering the entire system. It is nontrivial to show that the endemic equilibrium E_e is locally stable. This requires the Routh-Hurwitz criteria applied to the Jacobian matrix evaluated at E_e for all 10 differential equations. Therefore, Theorem 6 is incorrect. Alternately given specific parameter values the eigenvalues of the Jacobian matrix can be computed

In several figures, either the wild or domestic animals approach the dfe. Why? The term "reservoir" implies that the basic reproductive number $\beta_w b_w/(m_w(\gamma_w + m_w)) > 1$ (typo in (14)). Methods for parameter sensitivity, such as Latin hypercube sampling and partial rank correlation coefficient, may be useful. The parameters p_d, μ, and p_h are important, as they connect the three populations.

The differential equations can be written in the text, rather than in a Table. There are three reproductive numbers, one for each population. A suggestion is to define R_0 in (14) as follows:

$$R_0 = \max\{R_0^w, R_0^d, R_0^h\}.$$

Then the three reproductive numbers can be given for the figures.
