# Supplementary material for: Mathematically modeling spillovers of an emerging infectious zoonosis with an intermediate host
Source: PLoS One. 2020 Aug 26;15(8):e0237780. doi: 10.1371/journal.pone.0237780 (PMC7449412; doi:10.1371/journal.pone.0237780)
Supplement: Attachment — Submitted filename: Response to Reviewers.pdf [file pone.0237780.s004.pdf]

Response to Reviewers

Katherine Royce and Feng Fu

March 6, 2020

We have incorporated ideas from Plowright et al. 2017, Childs et al. 2019, and Washburne et al. 2019. Per Faust et al. 2017, we have included a suggestion to incorporate logistic growth of each host in future research, but our deterministic model is not sensitive to disease-induced mortality (since, as noted in the Appendix, any infection in an upstream host will spill over to downstream ones even if the epidemic dies out among that particular species). A stochastic model, which we suggest as another possible extension of our work, may retain the sensitivity to disease-induced mortality that this model lacks; however, this model was intended to provide a preliminary basis for quantitative investigation of zoonoses with intermediate hosts, and so the question of the effects of disease-induced mortality is beyond the scope of the current paper. We have incorporated Figures 4 and 5, heat maps with p_h and p_d as the axes and I_h as the surface, for four values of μ. We performed this experiment for $\beta_h = 0.078$ and for $\beta_h = 0$. We have shortened the introduction to 4 paragraphs and conformed to a more traditional manuscript format, following the suggested structure. Further, we have combined the original figures into 5 final versions, consolidating our findings. We have revised our description of zoonotic pathogens to match the behavior being discussed in each instance. We have deleted the discussions of dengue (L174-179) and of applications (L248-252), as well as Tables 5 and 6, and moved a mathematical proof of our results to an appendix. Finally, we have included the Matlab code used to produce our results as a supplemental file.
