# Supplementary material for: Mathematically modeling spillovers of an emerging infectious zoonosis with an intermediate host
Source: PLoS One. 2020 Aug 26;15(8):e0237780. doi: 10.1371/journal.pone.0237780 (PMC7449412; doi:10.1371/journal.pone.0237780)
Supplement: Attachment — Submitted filename: Response_to_Reviewers (2).pdf [file pone.0237780.s005.pdf]

Response to Reviewers

Katherine Royce and Feng Fu

May 24, 2020

We respond to the following points from the Academic Editor:

1. Journal Requirements: we have ensured that the figures are properly labeled and that the manuscript complies with the PLoS ONE LaTeXtemplate. We have deleted the supplementary writing and included the relevant analysis in the Methods section, and have ensured that our supplementary code complies with formatting requirements.

We respond to Reviewer 1's feedback:

1. We have incorporated ideas from Plowright et al. 2017, Childs et al. 2019, and Washburne et al. 2019, highlighting the differences between the conceptual models of spillover dynamics that do not include disease dynamics in nonhuman species and our model, which explicitly accounts for disease mutation in a nonhuman host.

2. Per Faust et al. 2017, we have included a suggestion to incorporate logistic growth of each host in future research, but our deterministic model is not sensitive to disease-induced mortality (since any infection in an upstream host will spill over to downstream ones even if the epidemic dies out among that particular species). Our goal is to provide a conceptual framework for intermediate host transmission in many contexts, and we have thus kept the simpler assumption of constant birth and death rates in order to make the model accessible in many different contexts. A stochastic model, which we suggest as another possible extension of our work, may retain the sensitivity to disease-induced mortality that this model lacks; however, this model was intended to provide a preliminary basis for quantitative investigation of zoonoses with intermediate hosts, and so the question of the effects of disease-induced mortality is beyond the scope of the current paper.

3. We have incorporated Figures 4 and 5, heat maps with p_h and p_d as the axes and I_h as the surface, for four values of μ, giving a more complete explanation of where in parameter space the proportion of infected humans is maximized. Further, we performed this experiment for $\beta_h = 0.078$ and for $\beta_h = 0$, distinguishing between a human-to-human transmissible pathogen and one that cannot be transmitted among humans.

4. We have shortened the introduction to 4 paragraphs and conformed to a more traditional manuscript format, following the suggested structure. Further, we have combined the original figures into 5 final versions, consolidating our findings.

5. We have clarified which word we mean depending on context, revising our description of zoonotic pathogens to match the behavior being discussed in each instance.

6. We have deleted the discussions of dengue (L174-179) and of applications (L248-252), and streamlined the mathematical proofs of our results.

7. We have deleted Tables 5 and 6, replacing them with the heatmaps described in item 3.

8. We have included the Matlab code used to produce our results as a supplemental file.

We respond to Reviewer 2's feedback:

1. We have included a formula for R_0 in the model and noted the similarities to multigroup models, as well as including code to calculate R_0 given specific parameter values. In addition, we have included a reference to the Routh-Hurwitz criteria to ensure completeness of the analysis.

2. We have corrected the typo in (14) and clarified the importance of p_d, p_h, and μ. While we considered Latin hypercube sampling, in particular, the aim of this paper is to introduce a new type of model, with the goal of prompting further research, and we have thus kept the analysis as simple as possible.

3. We have clarified (lines 161-181) the distinction between intracompartmental reproductive numbers and the global reproductive number, explaining why the pathogen can fade in animal species while reaching an endemic equilibrium in humans. We have also noted that this behavior may cause the wild species not to be a 'reservoir host' in the true sense of the term (line 176).

4. While we have kept the differential equations in a table, following conventions in mathematical biology, we have explicitly stated R_0 on line 160 and given a numerical R_0 for each of the figures.

We thank the reviewers for their insightful commentary.
