# Supplementary material for: Mathematically modeling spillovers of an emerging infectious zoonosis with an intermediate host
Source: PLoS One. 2020 Aug 26;15(8):e0237780. doi: 10.1371/journal.pone.0237780 (PMC7449412; doi:10.1371/journal.pone.0237780)
Supplement: Attachment — Submitted filename: review_PONE_2020.pdf [file pone.0237780.s006.pdf]

Review of
"Mathematical modeling spillovers of an emerging infectious zoonosis with an intermediate host"
PLOS ONE

The new manuscript has been completely revised, eliminating the Theorems and Proofs in the Methods and Analysis sections and condensing the material into a new shortened Methods section. The figures have also been revised with the exception of the compartmental diagram and Figure 2. Heat maps replace the curves showing temporal dynamics. The maps show the proportion of infected human population as three intercompartmental parameters are varied. The revision is much improved over the original manuscript. There are a few problems with the revision that need to be addressed.

1. Many statements begin with the citation number, instead of the authors names. See, for example, statements in the Introduction:
(9) blames a desire to view zoonoses...
(45) considers the risk...
(19) and (23) conceptualize...
(11) cites two barriers,...
etc.

2. The inequality in Table 4 for Domestic population S_d^* does not make it clear that the endemic equilibrium value for S_d^* is unique. The verification of uniqueness of $S_d^* > 0$ can be put in an Appendix.

3. The statement "R_0 as a measure of the global stability of the epidemic" is misleading. Nothing has been verified about global stability of the disease-free equilibrium. The reference [37] provides local stability criteria for the disease-free equilibrium. Suggestions are to eliminate any mention of "global R_0" or verify global stability of the disease-free equilibrium. Please see the paper by Shuai and van den Driessche in SIAM J. Appl. Math. 2013.

4. Replace "pathenogenic" with pathogenic. Typographical error "he" in Figure 3 caption.

5. Figure 2 caption or Table 5 has errors. Caption of Figure 2 gives the value $R_0 = 2.0871$ but based on the values in Table 5, $R_0^h = 1.0227$ and $R_0^w = R_0^d = 0.4493 > R_0^{dm}$. Therefore, $R_0 = 1.0227$.

6. The values used for β_d, β_w and other parameters for the heat maps need to be stated in the text (Figures 4 and 5). It was only by looking at the Matlab programs that I found their values. The values of $\beta_d = \beta_w$ inTable 5 are multiplied by 5, e.g., $\beta_d = 0.0118(5) = \beta_h$, then $R_0^w = R_0^d = 0.4493(5) = 2.2463$ which agrees with the value for R_0 in the captions of Figures 4 and 5.

7. The heat map axes are blurred.

8. In the Discussion, when referring to β_i, γ_i and m_i versus and p_d, p_h and μ it is easier to understand if the phrases intracompartmental versus intercompartmental parameters are used instead of first or second category or second parameters.

9. The first statement in the Discussion is not correct. The disease-free equilibrium is locally stable if $R_0 < 1$ and R_0 does not depend on μ, p_d or p_h. Was the local stability of the endemic equilibrium (EE) verified in the Matlab codes for all EE values generated from the p_d, p_h and μ values in the heat maps?

10. The following statement is misleading: "We prove that it is only these second parameters, and the initial proportional of wild animals that have the potential to alter the global dynamics of

the three-species system to a disease-free equilibrium." A suggestion is to replace the underlined phrase prove that it is only these second parameters, with "illustrate through several numerical examples that the intercompartmental parameters,".

11. There is mention of an Appendix but I did not find any Appendices.

12. There is mention of section 4.1 but there is no section 4.1.

13. The Discussion and Conclusion are long and they can be shortened. It is clear from the model formulation and the nice compartmental diagram that p_d, p_h and μ are the parameters that link all three species. If an infection begins within the wild animal reservoir, $I_w(0) > 0$, then in order to cause a human infection all three parameters must be positive. Prevention of a new zoonosis depends on breaking one or more of these links.
