# Supplementary material for: Mathematically modeling spillovers of an emerging infectious zoonosis with an intermediate host
Source: PLoS One. 2020 Aug 26;15(8):e0237780. doi: 10.1371/journal.pone.0237780 (PMC7449412; doi:10.1371/journal.pone.0237780)
Supplement: Attachment — Submitted filename: Response_to_Reviewers.pdf [file pone.0237780.s007.pdf]

Response to Reviewers

Katherine Royce and Feng Fu

July 27, 2020

We respond to Reviewer 1's feedback:

1. We have discussed the limitations of our model in lines 159-163, and included more discussion of logistic growth in the Discussion.
2. We have toned down L21, reworded L46 (now L47) as suggested, and rewritten L75 (now L83) to capture the nuance of multihost modeling.
3. The units of time in the parameterization are in days; we clarify here and throughout the paper.
4. We agree that incorporating logistic growth and pathogen virulence is a necessary continuation of this research; however, we have kept the model as simple as possible to facilitate adaptations to specific diseases for others. As a result, this model is best suited to pathogens with low host mortality and the early stage of a zoonotic epidemic with a delay between infection and mortality. We have added this clarification in the first item above, and included lines 302-306 in the Discussion to emphasize this limit.
5. Figure 2 has been revised to standardize the y-axes and clarify the axis labels.
6. Figure 3 has been revised to standardize the y-axis and clarify the axis labels. In addition, we have revised the figure to reflect the impact of β_h on I_h: as is more clear now, varying β_h causes I_h to change from 0.185 to 0.22, although this is a less drastic change than β_d.
7. Figures 4 and 5 have been revised to reflect the $\mu-$range in the literature, clarify the axes, and label the surface. (While Matlab does not allow colorbar labeling in heatmaps, the figure captions state the surface values.)

We respond to Reviewer 2's feedback:

1. We have added author names to the in-text citations.
2. We have added an appendix proving the uniqueness of the equilibrium value for S_d^*, which follows from there only being one intersection point within population parameters.

3. We have deleted references to a "global" R_0 and cited the suggested paper in L181.
4. We have corrected the typographical errors.
5. The code used to produce Figure 2 erroneously used $\gamma_h = 0.4$; with $\gamma_h = 0.091$ as in Table 5, the values for R_0 agree.
6. We have edited the captions of Figures 4 and 5 to state the parameter values used.
7. We have revised Figures 4 and 5 to clarify the axes.
8. We have implemented the suggested change in phrasing.
9. We have rephrased the first sentence of the Discussion to refocus on the spillover potenetial of the pathogen, and have added code to verify the stability of the equilibria for each value shown in the heatmaps (see `heatmap_generator.m`).
10. We have replaced the phrase in 261-262 as suggested.
11. We have deleted the reference to an Appendix.
12. We have deleted the reference to Section 4.1.
13. We have shortened the Discussion from 103 lines to 76 lines and the Conclusion from 46 lines to 29 lines, deleting the paragraph formerly on lines 381-396.

We have also removed the unused references previously included in revised portions. We thank the reviewers for their insightful commentary.
