# Supplementary material for: Mathematically modeling spillovers of an emerging infectious zoonosis with an intermediate host
Source: PLoS One. 2020 Aug 26;15(8):e0237780. doi: 10.1371/journal.pone.0237780 (PMC7449412; doi:10.1371/journal.pone.0237780)
Supplement: Attachment — Submitted filename: Response_to_Reviewers.pdf [file pone.0237780.s008.pdf]

Response to Reviewers

Katherine Royce and Feng Fu

July 31, 2020

We have revised lines 162 to reference the 2009 pandemic influenza instead of Covid-19 and Ebola, and have updated lines 303-306 to reflect this change. We have also removed the reference to the revision process on line 302. Further, we have reordered the references in Table 1 so that the sources are cited in numerical order.

We thank the reviewers for their feedback.
